# Supplementary material for: Polyphenols in Cereals: State of the Art of Available Information and Its Potential Use in Epidemiological Studies
Source: Nutrients. 2024 Jul 6;16(13):2155. doi: 10.3390/nu16132155 (PMC11243113; doi:10.3390/nu16132155)
Supplement: Supplementary file 1 [file nutrients-16-02155-s001.zip › nutrients-3061269-supplementary.pdf]

Table S1. Phenolic composition and levels in cereals and in cereal-based products

Cereals/ cereal products	Polyphenol class	Compound (mg/100g, edible portion)	Technique	Data source: Phenol-explorer	Data source: USDA	Reference
Cereal grains and flour						
Wheat (*Triticum aestivum* L. ssp. *aestivum*), whole grain flour	Flavones	Apigenin 6,8-C-arabinoside-C-glucoside (30.47); Apigenin 6,8-C-galactoside-C-arabinoside (46.82)	HPLC	√		*Asenstorfer et al. (2006)*
	Hydroxycinnamic acids	Caffeic acid (0.04); Ferulic acid (0.15); p-Coumaric acid (0.03); Sinapic acid (0.01)	HPLC	√		*Weidner et al. (1999)*
	Alkylphenols	5-Heneicosenylresorcinol (16.78)	HPLC	√		*Mullin et al. (1992)*
		5-Heneicosenylresorcinol (19.29);	GC-MS			*Gohil et al. (1988)*
		5-Heptadecylresorcinol (1.80);	HPLC			*Mullin et al. (1992)*
		5-Nonadecenylresorcinol (2.80);	HPLC			*Mattila et al. (2005)*
		5-Nonadecylresorcinol (14.51);	HPLC			*Mullin et al. (1992)*
		5-Pentacosenylresorcinol (1.71);	HPLC			*Mullin et al. (1992)*
		5-Pentacosylresorcinol (0.59);	HPLC			*Mattila et al. (2005)*
		5-Pentadecylresorcinol (0.18);	HPLC			*Ross et al. (2003)*
		5-Tricosenylresorcinol (3.43);	HPLC			*Mullin et al. (1992)* *Ross et al. (2003)*

		5-Tricosylresorcinol (3.05)	HPLC			
Wheat, purple	Anthocyanidins	Cyanidin (11.07); Delphinidin (3.20); Malvidin (4.02); Pelargonidin (3.41); Peonidin (1.81); Petunidin (2.34)	UPLC/MS/MS		⌂	*Hosseinian et al. (2008)*
Wheat (*Triticum aestivum* L. ssp. *aestivum*), refined flour	Hydroxybenzoic acids	Syringic acid (0.37)<br>Vanillic acid (0.32)	GLC-MS	√		*Sosulski et al. (1982)*
	Hydroxycinnamic acids	Ferulic acid (5.31)<br>Trans-Ferulic acid (5.72)	GLC-MS	√		*Sosulski et al. (1982)*
	Lignans	Lariciresinol (0.18)<br>Matairesino (2.14e-04)<br>Pinoresinol (9.00e-03)<br>Secoisolariciresinol (0.02)	LC-MS	√		*Milder et al. (2005)*<br><br>*Penalvo et al. (2008)*
Hard wheat (*Triticum durum* Desf.), whole grain flour	Hydroxycinnamic acids	Ferulic acid (72.21)	HPLC	√		*Lempereur et al. (1997)*
	Alkylphenols	5-Heneicosylresorcinol (23.82)<br>5-Heptadecylresorcinol (0.24)<br>5-Nonadecylresorcinol (5.90)<br>5-Pentacosylresorcinol (2.82)<br>5-Pentadecylresorcinol (0.45)<br>5-Tricosylresorcinol (8.69)	HPLC	√		*Landberg et al. (2006)*
Hard wheat (*Triticum durum* Desf.), semolina	Hydroxycinnamic acids	5-5'-Dehydrodiferulic acid (4.49)<br>5-8'-Benzofuran dehydrodiferulic acid (8.23)<br>5-8'-Dehydrodiferulic acid (6.13)<br>8-O-4'-Dehydrodiferulic acid (11.02)<br>Cis-Ferulic acid (0.11)	HPLC	√		*Lempereur et al. (1998)*<br>*Peyron et al. (2002)*

		Ferulic acid (64.04)<br>Sinapic acid (0.33)<br>Trans-Ferulic acid (1.03)				
	Lignans	Secoisolariciresinol (2.00e-03)	LC-MS	√		*Kuhnle et al. (2009)*
Maize (*Zea mays* L.), whole grain	Hydroxycinnamic acids	Ferulic acid (0.53)	HPLC	√		*Adom et al. (2002)*
Maize (*Zea mays* L.), refined flour	Hydroxybenzoic acids	4-Hydroxybenzoic acid (0.02)<br>Protocatechuic acid (0.09)<br>Syringic acid (0.09)<br>Vanillic acid (0.08)	GLC-MS	√		*Sosulski et al. (1982)*
	Hydroxycinnamic acids	Caffeic acid (0.04)<br>Cis-Ferulic acid (0.06)<br>p-Coumaric acid (0.33)<br>Trans-Ferulic acid (0.34)	GLC-MS	√		*Sosulski et al. (1982)*
	Hydroxyphenylacetic acids	4-Hydroxyphenylacetic acid (0.09)	GLC-MS	√		*Sosulski et al. (1982)*
Barley (*Hordeum vulgare* L. ssp. *vulgare)*, whole grain flour	Flavanols	(+)-Catechin (1.23)<br>(+)-Catechin (1.23)<br>Procyanidin dimer B3 (10.90)<br>Prodelphinidin dimer B3 (23.07)	HPLC	√<br><br>√	⌂	*Madigan et al. (1994)* Holtekjølen et al. (2006)
	Alkylphenols	5-Heneicosylresorcinol (1.18)<br>5-Heptadecylresorcinol (0.19)<br>5-Nonadecylresorcinol (0.71)<br>5-Pentacosylresorcinol (1.65)<br>5-Pentadecylresorcinol (0.01)<br>5-Tricosylresorcinol (0.77)	HPLC	√		*Ross et al. (2003)*

Oat (*Avena sativa* L.), whole grain flour	Hydroxybenzoic acids	4-Hydroxybenzoic acid (0.45)<br>Vanillic acid (0.27)	HPLC	√	*Emmons et al. (1999)*
	Hydroxycinnamic acids	Avenanthramide 2c (3.85)<br>Avenanthramide 2f (2.65)<br>Avenanthramide 2p (2.70)<br>Avenanthramide K (1.95)<br>Caffeic acid (0.16)<br>Ferulic acid (0.19)<br>p-Coumaric acid (0.16)<br>Sinapic acid (0.04)	HPLC	√	*Dimberg et al. (1996)*<br>*Emmons et al. (1999)*
	Hydroxybenzaldehydes	4-Hydroxybenzaldehyde (0.12)<br>Vanillin (0.21)	HPLC	√	*Dimberg et al. (1996)*<br>*Emmons et al. (1999)*
Oat (*Avena sativa* L.), refined flour	Hydroxybenzoic acids	4-Hydroxybenzoic acid (0.06)<br>Protocatechuic acid (0.04)<br>Syringic acid (0.20)<br>Vanillic acid (0.06)	GLC-MS	√	*Sosulski et al. (1982)*
	Hydroxycinnamic acids	Avenanthramide 2f (8.12)<br>Caffeic acid (0.04)<br>p-Coumaric acid (0.06)<br>Trans-Ferulic acid (0.21)	GLC-MS	√	*Sosulski et al. (1982)*
	Hydroxyphenylacetic acids	4-Hydroxyphenylacetic acid (0.03)	GLC-MS	√	*Sosulski et al. (1982)*
Rice (*Oryza sativa* L.) whole grain	Hydroxybenzoic acids	4-Hydroxybenzoic acid (0.07)<br>Syringic acid (0.03)<br>Vanillic acid (0.13)	GC	√	*Kato et al. (1983)*
	Hydroxycinnamic acids	Caffeic acid (0.05)<br>Ferulic acid (0.10)<br>p-Coumaric acid (0.02)	GC	√	*Kato et al. (1983)*

Rice (*Oryza sativa* L.) parboiled and refined*	Hydroxybenzoic acids	4-Hydroxybenzoic acid (0.08/0.17*)<br>Syringic acid (0.01)<br>Vanillic acid (0.29/0.11*)	GC	√	*Kato et al. (1983)*
	Hydroxycinnamic acids	Caffeic acid (0.34)<br>Ferulic acid (0.17/0.02*)<br>p-Coumaric acid (0.19/0.11*)<br>Trans-Ferulic acid (0.21*)	GC	√	*Kato et al. (1983)*
Rye (*Secale cereale* L.), whole grain flour	Hydroxycinnamic acids	Caffeic acid (0.20)<br>Ferulic acid (0.45)<br>p-Coumaric acid (0.23)<br>Sinapic acid (0.21)	HPLC	√	*Weidner et al. (1999)*
	Alkylphenols	5-Heneicosylresorcinol (16.17)<br>5-Heptadecylresorcinol (15.11)<br>5-Nonadecenylresorcinol (3.55)<br>5-Nonadecylresorcinol (20.41)<br>5-Pentacosylresorcinol (5.21)<br>5-Pentadecylresorcinol (1.64)<br>5-Tricosylresorcinol (7.01)	HPLC	√	*Mattila et al. (2005)*<br>*Ross et al. (2003)*
Rye (*Secale cereale* L.), refined flour	Alkylphenols	5-Heneicosylresorcinol (2.40)<br>5-Heptadecylresorcinol (2.13)<br>5-Nonadecylresorcinol (3.11)<br>5-Pentacosylresorcinol (0.44)<br>5-Tricosylresorcinol (0.80)	HPLC	√	*Chen et al. (2004)*
Sorghum (*Sorghum bicolor* L.), whole grain	Hydroxybenzoic acids	Protocatechuic acid (2.55)	HPLC	√	*Beta et al. (1999)*

Sorghum, grain, red	Flavanones	Eriodictyol (0.29)<br>Naringenin (1.67)	HPLC		⌂	*Dykes et al. (2009)*
	Flavones	Apigenin (2.54)<br>Luteolin (3.93)	HPLC		⌂	*Dykes et al. (2009)*
Bread						
Bread, wheat, whole grain flour	Alkylphenols	5-Heneicosylresorcinol (10.87)<br>5-Heptadecylresorcinol (1.53)<br>5-Nonadecylresorcinol (9.57)<br>5-Pentacosylresorcinol(0.89)<br>5-Tricosylresorcinol (1.85)	HPLC	√		*Ross et al. (2003)*
Bread, wheat, refined flour	Lignans	Lariciresinol (0.01)<br>Matairesinol (1.23e-03)<br>Pinoresinol (0.01)<br>Secoisolariciresinol (7.19e-03)<br>Syringaresinol (0.04)	MS	√		*Milder et al. (2005)*<br>*Thompson et al. (2006)*
Bread, rye, whole grain flour	Hydroxybenzoic acids	Vanillic acid (0.57)	HPLC	√		*Mattila et al. (2002)*
	Hydroxycinnamic acids	Caffeic acid (0.77)<br>Ferulic acid (3.90)	HPLC	√		*Mattila et al. (2002)*
	Alkylphenols	5-Heneicosylresorcinol (14.63)<br>5-Heptadecylresorcinol (14.05)<br>5-Nonadecenylresorcinol (2.30)<br>5-Nonadecylresorcinol (20.03)<br>5-Pentacosylresorcinol (4.64)<br>5-Tricosylresorcinol (6.29)	HPLC	√		*Ross et al. (2003)*
Breakfast cereals						

Breakfast cereals, bran	Alkylphenols	5-Heneicosenylresorcinol (81.52)<br>5-Heneicosylresorcinol (92.76)<br>5-Heptadecylresorcinol (8.93)<br>5-Nonadecylresorcinol (48.31)<br>5-Pentacosenylresorcinol (7.84)<br>5-Pentacosylresorcinol (7.13)<br>5-Tricosenylresorcinol (19.55)<br>5-Tricosylresorcinol (19.62)	HPLC	√	*Mullin et al. (1992)*
Breakfast cereals, corn	Lignans	Matairesinol (1.67e-03)<br>Secoisolariciresinol (5.50e-03)	LC-MS	√	*Kuhnle et al. (2009)*
Breakfast cereals, muesli	Alkylphenols	5-Heneicosylresorcinol (5.71)<br>5-Heptadecylresorcinol (1.54)<br>5-Nonadecylresorcinol (3.99)<br>5-Pentacosylresorcinol (0.61)<br>5-Tricosylresorcinol (1.41)	HPLC	√	*Chen et al. (2004)*
Breakfast cereals, oat, wholemeal	Lignans	Matairesinol (0.06)<br>Secoisolariciresinol (0.02)	LC-MS	√	*Kuhnle et al. (2009)*
Pasta					
Pasta, whole grain	Alkylphenols	5-Heneicosylresorcinol (11.94)<br>5-Heptadecylresorcinol (0.25)<br>5-Nonadecylresorcinol (3.83)<br>5-Pentacosylresorcinol (1.46)	HPLC	√	*Landberg et al. (2006)*

5-Tricosylresorcinol (4.37)

Data source: Phenol Explorer √; USDA vers. 3.2 ⌂.

Literature cited in the Table S1:

97) Adom K.K., Liu R.H. (2002) Antioxidant activity of grains. *Journal of Agricultural and Food Chemistry* **50**:6182-6187.
98) Asenstorfer R.E., Wang Y., Mares D.J. (2006) Chemical structure of flavonoid compounds in wheat (Triticum aestivum L.) flour that contribute to the yellow colour of Asian alkaline noodles. *Journal of Cereal Science* **43**:108-119.
99) Beta T., Rooney L.W., Marovatsanga L.T., Taylor J.R.N. (1999) Phenolic compounds and kernel characteristics of Zimbabwean sorghums. *Journal of the Science of Food and Agriculture* **79**:1003-1010.
100) Chen Y., Ross A.B., Aman P., Kamal-Eldin A. (2004) Alkylresorcinols as markers of whole grain wheat and rye in cereal products. *Journal of Agricultural and Food Chemistry* **52**:8242-8246
101) Dimberg L.H., Molteberg E.L., Solheim R., Frohlich W. (1996) Variation in oat groats due to variety, storage and heat treatment. I. Phenolic compounds. *Journal of Cereal Science* **24**:263-272.
102) Dykes, l., Seitz, L. M., Rooney, W. L., and Rooney, L. W. (2009) Flavonoid composition of red sorghum genotypes. *Food Chemistry* **116**:313-317.
103) Emmons C.L., Peterson D.M., Paul G.L. (1999) Antioxidant capacity of oat (Avena sativa L.) extracts. 2. In vitro antioxidant activity and contents of phenolic and tocol antioxidants. *Journal of Agricultural and Food Chemistry* **47**:4894-4898.
104) Gohil S., Petterson D., Salomonsson A.-C., Aman P. (1988) Analysis of alkyl- and alkenylresorcinols in Triticale, wheat and rye. *Journal of the Science of Food and Agriculture* **45**:43-52.105) Holtekjølen, A. K., Kinitz, C., and Knutsen, S. H. (2006) Flavanol and bound phenolic acid contents in different barley varieties. J. Agric. Food Chemistry **54**:2253-2260.
106) Hosseinian, F. S., Li, W. and Beta, T. (2008) Measurement of anthocyanins and other phytochemicals in purple wheat. Food Chemistry **109**:916-924.
107) Kato H., Ohta T., Tsugita T., Hosaka Y. (1983) Effect of parboiling on texture and flavor components of cooked rice. *Journal of Agricultural and Food Chemistry* **31**:818-823.
108) Kuhnle G.G.C., Dell'Aquila C., Aspinall S.M., Runswick S.A., Mulligan A.A., Bingham S.A. (2009) Phytoestrogen content of cereals and cereal-based foods consumed in the UK. *Nutrition and Cancer* **61**:302-309.
109) Landberg R., Kamal-Eldin A., Andersson R., Aman P. (2006) Alkylresorcinol content and homologue composition in durum wheat (Triticum dururm) kernels and pasta products. *Journal of Agricultural and Food Chemistry* **54**:3012-3014.
110) Lempereur I., Surget A., Rouau X. (1998) Variability in dehydroferulic acid composition of durum wheat (Triticum durum Desf.) and distribution in milling fractions. *Journal of Cereal Science* **28**:251-258.
111) Lempereur I., Rouau X., Abecassis J. (1997) Genetic and agronomic variation in arabinoxylan and ferulic acid contents of durum wheat (Triticum durum L.) grain and its milling fractions. *Journal of Cereal Science* **25**:103-110.
112) Madigan D., McMurrough I. (1994) Determination of proanthocyanidins and catechins in beer and barley by high-performance liquid chromatography with dual-electrode electrochemical detection. *Analyst* **119**:863-868.

113) Mattila P., Pihlava J.-M., Hellstrom J. (2005) Contents of phenolic acids, alkyl- and alkenylresorcinols, and avenanthramides in commercial grain products. *Journal of Agricultural and Food Chemistry* **53**:8290-8295.
114) Milder I.E.J., Arts I.C.W., Van de Putte B., Venema D.P., Hollman P.C.H. (2005) Lignan contents of Dutch plant foods: a database including lariciresinol, pinoresinol, secoisolariciresinol and matairesinol. *British Journal of Nutrition* **93**:393-402
115) Mullin W.J., Emery P.H. (1992) Determination of alkylresorcinols in cereal-based foods. *Journal of Agricultural and Food Chemistry* **40**:2127-2130.
116) Penalvo J.L., Adlercreutz H., Uehara M., Ristimaki A., Watanabe S. (2008) Lignan content of selected foods from Japan. *Journal of Agricultural and Food Chemistry* **56**:401-09.
117) Peyron S., Surget A., Mabile, F., Autran J.C., Rouau X., Abecassis J. (2002) Evaluation of tissue dissociation of durum wheat grain (Triticum durum Desf.) generated by the milling process. *Journal of Cereal Science* **36**:199-208.
118) Ross A.B., Shepherd M.J., Schupphaus M., Sinclair V., Alfaro B., Kamal-Eldin A., Aman P. (2003) Alkylresorcinols in cereals and cereal products. *Journal of Agricultural and Food Chemistry* **51**:4111-4118
119) Sosulski F., Krygier K., Hogge L. (1982) Free, esterified, and insoluble-bound phenolic acids. 3. Composition of phenolic acids in cereal and potato flours. *Journal of Agricultural and Food Chemistry* **30**:337-340.
120) Thompson L.U., Boucher B.A., Liu Z., Cotterchio M., Kreiger N. (2006) Phytoestrogen content of foods consumed in Canada, including isoflavones, lignans, and coumestan. *Nutrition and Cancer* **54**:184-201.
121) Weidner S., Amarowicz R., Karamac M., Dabrowski G. (1999) Phenolic acids in caryopses of two cultivars of wheat, rye and triticale that display different resistance to pre-harvest sprouting. *European Food Research and Technology* **210**:109-113.
